# Address sustainability risks in health insurance funds: generational actuarial balance and intergenerational equity perspective

**DOI:** 10.3389/fpubh.2025.1641233

**Published:** 2025-08-08

**Authors:** Yi Qin, Wenfang Ji

**Affiliations:** School of Insurance and China Institute for Actuarial Science, Central University of Finance and Economics, Beijing, China

**Keywords:** health insurance systems, sustainability risks, generational actuarial balance, intergenerational equity, China’s employee basic health insurance

## Abstract

**Background:**

Public health insurance systems worldwide face growing sustainability risks due to aging populations and rising medical costs. China’s employee basic health insurance (CEBHI) system is particularly vulnerable, with concerns about generational actuarial imbalances and intergenerational inequities threatening its long-term viability.

**Methods:**

This study constructs an intergenerational accounting framework for the CEBHI system, analyzing its sustainability from the perspectives of generational actuarial balance and intergenerational equity. We evaluate the impact of potential policy adjustments, including delayed retirement age, retiree contributions, increased premium rates, and reduced reimbursement rates.

**Results:**

The findings reveal severe generational actuarial imbalances and intergenerational inequities within the EBHI system. While individual policy measures—such as delaying retirement, introducing retiree contributions, raising premiums, or lowering reimbursement rates—can partially mitigate sustainability risks, none alone achieves both actuarial balance and intergenerational equity. Policy coordination is essential. Notably, delayed retirement has a limited impact, whereas retiree contributions are critical in all effective policy combinations.

**Conclusion:**

To ensure the long-term sustainability of China’s health insurance fund, the government must adopt synergistic policy combinations, prioritizing reforms in retirement age and retiree contribution policies. Isolated adjustments are insufficient; integrated strategies are necessary to address systemic risks.

## 1 Introduction

Public health insurance systems are crucial in safeguarding public health and covering basic medical expenses. By providing coverage for insured individuals, these systems help people cope with the financial burdens of illness or accidental injury, ensuring that people have access to necessary treatment. Countries worldwide have established health insurance systems based on their socio-economic conditions, the distribution of medical resources, and the health needs of their citizens (1). However, challenges such as an aging population and continuously rising healthcare costs pose unprecedented sustainability risks to health insurance systems. How to address these risks and ensure the long-term stability of health insurance systems has become a key issue of widespread concern in the international community (2–8).

Countries have taken various measures to address the sustainability risks of public health insurance schemes. For example, Germany and the Netherlands have pursued diversification of health insurance by introducing a supplementary insurance mechanism and promoting the participation of private insurance companies to share part of the financial pressure (9, 10). To address financial pressures from population aging, many pay-as-you-go pension systems have raised or plan to increase the statutory retirement age (11). China has begun to implement a delayed retirement policy, which has helped reduce the financial burden of health insurance (12). Countries like Germany and France require retirees to pay a percentage of health insurance premiums (13). In high-tax Nordic countries, broad-based income taxes and value added tax collectively provide the fiscal capacity to sustain universal health coverage (14). In addition, some countries have reduced the incidence of major diseases by implementing health promotion and public health programs, such as health screenings, vaccinations, and chronic disease management, thereby reducing the pressure on healthcare expenditures.

The current public health insurance response strategy focuses mainly on preventing risks to fund revenues and expenditures, which has achieved some success but lacks a systemic framework. The limitations of this single perspective are becoming increasingly evident: with an aging population and rising health-care costs, the system faces deeper intergenerational tensions (15). China’s Employee Basic Health Insurance (CEBHI) — a pay-as-you-go public health insurance program covering 371 million participants — serves as a pivotal case study for research on intergenerational burdens. The sustainability challenges confronting this system are fundamentally rooted in two interconnected structural contradictions:

The first dimension concerns the issue of individual actuarial imbalance. Due to the current policy exempting retirees from premium contributions and contribution rates still anchored in the 1990s demographic structure and healthcare cost levels, population aging and rapidly growing medical expenses inevitably create severe lifecycle imbalances between contributions and benefits (16). This actuarial gap is termed implicit debt in academic literature (17).

The second dimension involves the issue of intergenerational inequity. As the worker-to-retiree ratio continues to deteriorate, declining from 3.09:1 in 2000 to 2.71:1 in 2023 according to National Bureau of Statistics data, younger generations are now required not only to bear their own healthcare insurance responsibilities but also to assume the costs of intergenerational transfers for retirees through the pay-as-you-go system (18). Failure to effectively resolve such intergenerational pressures will directly threaten the sustainability of the system.

Current research exhibits three distinct limitations: First, the majority of literature remains confined to measuring the scale of funding gaps (19, 20) without uncovering the structural mechanisms driving gap formation. Second, while a subset of scholars has addressed intergenerational equity concerns (18, 21), their analyses remain largely confined to qualitative discourse and lack robust validation through systematic quantitative methodologies. Most critically, existing studies have yet to establish a cohesive analytical framework that bridges individual actuarial balance with intergenerational equity, leaving a fundamental gap in addressing systemic challenges.

Regarding the representativeness of CEBHI and existing research gaps, this study’s innovation lies in establishing a dual-constraint evaluation framework: actuarial balance constraint and intergenerational equity constraint. Specifically, each generation’s lifetime insurance contributions must balance with their healthcare benefits received, while simultaneously ensuring intergenerational equity in contribution-benefit distributions. The necessity of this dual consideration arises from the inherent policy dilemma: The presence of implicit debt in employee health insurance means that solely pursuing actuarial balance may shift debt burdens to future generations, exacerbating intergenerational inequity, whereas exclusive emphasis on intergenerational equity could inflate premiums beyond beneficiaries’ actual healthcare gains, triggering individual actuarial imbalances and financial losses. Consequently, only through mutually enforcing actuarial balance and intergenerational equity constraints can we identify optimal policy adjustments for ensuring the healthcare system’s sustainability.

The unique value of CEBHI as a research subject stems from China’s ongoing intense demographic transition, compounded by cost rigidity induced healthcare expenditure growth from technological advancements. Its evolving intergenerational conflict pathways offer early policy warnings and reform paradigms for other nations. This study will develop an intergenerational accounting model to quantitatively assess dual impacts of policy adjustments on actuarial balance and intergenerational equity, ultimately proposing reform pathways that balance financial sustainability with social acceptability.

The arrangement of the following sections is as follows: Section 2 introduces the CEBHI system and the research methodology; Section 3 focuses on model construction; Section 4 addresses parameter settings; Section 5 presents the calculation results of the intergenerational account values of the CEBHI; Section 6 examines the impact of policy changes on generational actuarial balance and intergenerational equity; finally, we conclude and make recommendations.

## 2 The CEBHI system and research method

The CEBHI system was created by implementing Document No. 44 [1998], making participation mandatory for all urban enterprises and employees. It consists of a pooled account for mutual assistance and individual accounts, with employers contributing 6% and employees contributing 2% of their salaries. Retirees are exempt from premiums but receive support from the pooled account. However, the system faces potential deficits due to aging populations and rising medical costs.

To address these challenges, the Chinese government has taken steps such as gradually increasing contribution rates and reallocating employer contributions to the pooled account starting in 2021. Additionally, a delayed retirement policy was introduced in 2025 to improve sustainability.

Despite these efforts, systematic theoretical support for these policies is lacking. This article studies the sustainability of the CEBHI fund through the perspectives of generational actuarial balance and intergenerational equity, proposing targeted policy recommendations to ensure long-term stability.

The intergenerational accounting method, proposed by Auerbach et al. (22), is used to assess the fiscal sustainability of such systems. Countries and international organizations have widely adopted this method for evaluating intergenerational equity. The paper innovatively combines this approach with an actuarial health insurance model, focusing on the long-term sustainability of the CEBHI fund. The paper creates indicators for generational actuarial balance and intergenerational equity by forecasting the insured population and estimating reimbursement costs.

Using this framework, the paper analyzes the impact of existing policies on generational balance and equity, simulates the effect of different policy scenarios, and provides evidence for policymakers to optimize future measures, ensuring the stability of the CEBHI system.

The CEBHI system, established under China’s 1998 Document No. 44, mandates participation from all urban enterprises and employees. It operates through a dual-account structure combining a social pooling account and individual accounts: employers contribute 6% of total payroll, while employees contribute 2% of their individual wages. Retirees are exempt from premium payments yet retain access to pooling account benefits. However, under the dual pressures of population aging and escalating healthcare costs, the system faces growing risks of actuarial deficit (19, 20).

To address healthcare fund sustainability challenges, the Chinese government has enacted a trilogy of policy responses: granting local governments discretionary power to calibrate contribution rates according to regional demographics; channeling all employer premiums exclusively into the pooling account since 2021; and implementing delayed retirement provisions effective 2025. Nevertheless, these measures remain theoretically fragmented. This study examines CEBHI fund sustainability through the dual lenses of individual actuarial balance and intergenerational equity, proposing targeted policy recommendations to ensure long-term institutional viability.

In terms of research methodology, this systemic analysis is grounded in the classical generational accounting literature. Seminal studies have assessed intergenerational equity by comparing generational account value ratios between current and future generations under fiscal policies, with disparity magnitudes ranging from 17 to 24% (22). Empirical studies demonstrate this analytical framework’s robust applicability in diverse policy evaluation contexts, with its methodological rigor thoroughly verified through cross-institutional validation studies (23).

As a fiscal policy analysis tool, this methodology establishes intergenerational budget constraints from the governmental perspective, stipulating that the present value of future government consumption must equal the combined present values of taxes paid by current residents during their remaining lifetimes, lifetime taxes from future residents, and existing net wealth, all while incorporating time value adjustments and productivity growth projections. Given that generational account values derive exclusively from net present value differentials between taxes and benefits (excluding public goods like national defense), their full lifecycle values remain inherently positive.

The healthcare insurance system’s distinctive nature, however, introduces methodological adaptation challenges: insured individuals’ lifetime contribution-benefit differentials may fluctuate between positive and negative values, fundamentally disrupting direct cross-generational comparability of account values. To resolve this constraint, we implement adaptive recalibration of intergenerational equity metrics: while retaining account value differentials as numerators, denominators are reconfigured as present values of current generations’ lifetime contributions to neutralize sign interference in intergenerational comparisons. Simultaneously, actuarial balance ratios are constructed as generational account value-to-lifetime contribution ratios, ensuring horizontal comparability across policy scenarios. This enhanced methodology enables systematic integration of classical theory with health insurance actuarial modeling. Through projections of insured population structures, economic parameters, and medical expenditure trends, we establish a dynamic evaluation index system for assessing intergenerational actuarial balance and equity within CEBHI system.

## 3 The intergenerational accounting framework

In pursuit of the goal of sustainability for the CEBHI fund, we construct an intergenerational accounting system. This system consists of four parts: first, it establishes the per capita intergenerational accounts for current generations based on the implementation process of the CEBHI; second, it sets up the intergenerational accounting constraints; third, it calculates the intergenerational account values for future generations based on the intergenerational account values of current generations and the accounting constraints; finally, it constructs indicators for generational actuarial balance and intergenerational equity.

### 3.1 Intergenerational accounting for current generations

The CEBHI fund is divided into a pooled fund and individual accounts. Since the funds entering individual accounts are at the individual’s discretion and do not have a mutual assistance function, this paper considers only the income and expenditure of the pooled fund when establishing the intergenerational accounts. According to the regulations in the CEBHI documents, the intergenerational account value $N_{i,t,k}$ for each generation equals the insured individual’s lifetime contributions minus the insurance benefits. These benefits include health insurance reimbursement and funds transferred from the pooled fund to the individual account after retirement (referred to as account transfers). The specific calculation process is shown in Equation 1.


(1) $$N_{i,t,k}=J_{i,t,k}-B_{i,t,k}-F_{i,t,k}$$

In this context, t is the base year for calculation, i∈{0,1} represents females and males respectively, $N_{i,t,k}$ is the intergenerational account value for insured individuals born in year k at year t, $J_{i,t,k}$ is the present value of the total average premiums paid by individuals born in year k in the future, $B_{i,t,k}$ is the present value of the total average health insurance reimbursement received by individuals born in year k in the future, and $F_{i,t,k}$ is the present value of the total average funds transferred to individual accounts for individuals born in year k. From Equation 1, it can be seen that intergenerational accounts measure the contributions of each generation to the CEBHI system.

The overall intergenerational account value $N_{t,k}$ is the weighted average of the intergenerational account values for males and females, as shown in Equation 2.


(2) $$N_{t,k}=\sum_{i=0}^{1}N_{i,t,k}\cdot R_{i,t,k}/\sum_{i=0}^{1}R_{i,t,k}$$

Where $R_{i,t,k}$ represents the number of insured individuals born in year k at year t, the overall medical contributions $J_{t,k}$ , health insurance reimbursement $B_{t,k}$ , and account transfers $F_{t,k}$ can similarly be derived.

#### 3.1.1 Present value of total average contribution


(3) $$J_{i,t,k}=\sum_{s=t}^{k+T_{i}}[\begin{array}{l} W_{i,t}(\prod_{j=t}^{s}(1+g_{j}))u{\left(1+r\right)}^{t-s}\\ \tau_{s}\prod_{j=t}^{s}(1-d_{i,j,k}) \end{array}],k\leq t-z$$

Where $W_{i,t}$ represents the gender-specific social average wage in year t, $g_{j}$ is the wage growth rate, u is the CEBHI contribution rate, $\tau_{s}$ is the proportion of the pooled fund in the fund’s income, r is the interest rate, $T_{i}$ is the retirement age for different genders, $d_{i,j,k}$ is the mortality rate for insured individuals born in year k in year j, z is the age at which an individual initially participates in the CEBHI, and k ≤ t - z indicates that residents born in year k have already entered the workforce by year t.

#### 3.1.2 Present value of total average health insurance reimbursement


(4) $$B_{i,t,k}=\sum_{s=t}^{k+D}[\begin{array}{l} M_{t}(\prod_{j=t}^{s}(1+g_{j}))\lambda_{i,s-k}\\ {\left(1+r\right)}^{t-s}\prod_{j=t}^{s}(1-d_{i,j,k}) \end{array}],k\leq t-z$$

Where $M_{t}$ is the average health insurance reimbursement amount in year t, $g_{j}$ is the growth rate of health insurance reimbursement amounts, and $\lambda_{i,t}$ is the gender-specific health insurance reimbursement weight for insured individuals aged t.

#### 3.1.3 Present value of total average account transfers


(5) $$F_{i,t,k}=\sum_{s=t}^{D}[Y_{t}(\prod_{j=t}^{s}(1+g_{j}))\varsigma {\left(1+r\right)}^{t-s}\prod_{j=t}^{s}(1-d_{i,j,k})]k\leq t-z\;$$

Where $Y_{t}$ is the average pension amount in year t, and *ς* is the proportion of the funds transferred from the pooled fund to the individual accounts of retired employees relative to the pension.

Equations 1–5 show that the intergenerational account values for each current generation can be calculated under specific parameter settings.

### 3.2 Intergenerational accounting constraints

The intergenerational budget constraint for CEBHI can be established using the ideas from intergenerational accounting. Specifically, this can be stated as the present value of all current intergenerational accounts, the present value of future generations’ intergenerational accounts, and the cumulative balance of CEBHI in the baseline year, summing to zero, as shown in Equation 6.


(6) $$\sum_{i=0}^{1}\sum_{k=z}^{D}N_{i,t,t-k}R_{i,t,t-k}+\sum_{i=0}^{1}\sum_{k=-z+1}^{+\infty }N_{i,t,t+k}R_{i,t,t+k}+W_{t}=0$$

Where $W_{t}$ represents the fund balance in year t , and D is the maximum lifespan. The first term on the left side of Equation 6 means the present value of the intergenerational accounts for the current generation. In contrast, the second term represents the present value of the intergenerational accounts for future generations.

From Equation 6, we can infer that an increase in the total present value of current intergenerational accounts indicates an increase in the current generation’s contribution to the CEBHI system. Consequently, future generations will contribute less to the CEBHI system. This demonstrates that the intergenerational budget constraint effectively reflects the zero-sum nature and the medium—to long-term actuarial balance of the CEBHI system.

### 3.3 Intergenerational account for future generations

According to Equation 6, the intergenerational accounts for future generations can be calculated under certain assumptions. For this purpose, this paper makes three assumptions: first, that the total burden is evenly distributed among future generations; second, that their respective future generations bear the burden for each gender; and third, that the changes in the burden for future generations are assumed to be in sync with economic growth.

The calculation formula for the intergenerational accounts of future generations of males and females, $\bar{N_{i}}$ , is shown in Equation 7.


(7) $$\begin{array}{l} \sum_{i=0}^{1}\sum_{k=z}^{D}N_{i,t,t-k}R_{i,t,t-k}+\sum_{i=0}^{1}\\ \sum_{k=-z+1}^{+\infty }[\begin{array}{l} \bar{N_{i}}(\prod_{j=t}^{t+k}(1+g_{j}))\\ R_{i,t,t+k}{\left(1+r\right)}^{-k} \end{array}]+W_{i,t}=0 \end{array}$$

The pooled account balance for males and females is given by $W_{i,t}=(\frac{W_{t}}{\sum_{i=0}^{1}\sum_{k=z}^{D}R_{i,t,t-k}})*\sum_{k=z}^{D}R_{i,t,t-k}$ .

The overall intergenerational account $\bar{N}\;$ is the weighted average of the intergenerational accounts for future generations of males and females, as shown in Equation 8.


(8) $$\bar{N}=\sum_{i=0}^{1}\sum_{k=z-1}^{+\infty }\bar{N_{i}}R_{i,t,t+k}/\sum_{i=0}^{1}\sum_{k=z-1}^{+\infty }R_{i,t,t+k}$$

### 3.4 Generational actuarial balance indicator and intergenerational equity Indicator

#### 3.4.1 Generational actuarial balance indicator

With the baseline set at the beginning of year t, the observed cohort consists of current members who are age z. For this cohort, initially enrolled in the CEBHI, the intergenerational account value $N_{i,t,t-z}$ equals the balance of the present value of lifetime contributions minus the present value of health insurance benefits. This reflects the contribution value of participants to the CEBHI over a complete life cycle under the current system.

The indicator $\Psi_{i}$ is constructed to measure the CEBHI’s generational actuarial balance, as shown in Equation 9.


(9) $$\Psi_{i}=N_{i,t,t-z}/J_{i,t,t-z}$$

When $\Psi_{i}>0$ , it indicates that the present value of a participant’s lifetime contributions is greater than that of CEBHI benefits.

When $\Psi_{i}<0$ , it indicates that the present value of a participant’s lifetime contributions is less than that of CEBHI benefits.

When $\Psi_{i}=0$ , it indicates that the present value of a participant’s lifetime contributions is equal to that of CEBHI benefits, achieving generational actuarial balance at the individual level. The closer the absolute value of $\Psi_{i}$ is to zero, the higher the degree of generational actuarial balance, with the absolute value of the generational actuarial balance indicator representing the degree of actuarial imbalance in the CEBHI system.

In this paper, the CEBHI account is considered to achieve generational actuarial balance when $|\Psi_{i}|\leq 0.2$ , meaning that the absolute value of the insured’s intergenerational account is less than 20% of their lifetime contributions.

#### 3.4.2 Intergenerational equity indicator

We develop an intergenerational equity indicator $\Phi_{i},$ defined in Equation 10.


(10) $$\Phi_{i}=(\bar{N}-N_{i,t,t-z})/J_{i,t,t-z}$$

The intergenerational equity indicator $\Phi_{i}$ represents the rate of change between the intergenerational accounts of future generations and those of the current cohort at the time of initial enrollment. This indicator assesses the fairness in the distribution of contributions and benefits between current and future generations within the CEBHI system.

When $\Phi_{i}>0$ , this indicates that future generations are expected to contribute more to the CEBHI system than the current generation.

When $\Phi_{i}<0$ , it indicates that future generations are anticipated to contribute less to the CEBHI system than the current generation.

When $\Phi_{i}=0$ , this indicates that the contribution of future generations to the CEBHI system is equal to that of the current generation. The closer the absolute value of $\Phi_{i}$ is to zero, the higher the degree of intergenerational equity between future and current generations. Thus, the absolute value of the Intergenerational Equity Indicator represents the degree of intergenerational inequity within the CEBHI system. In this paper, the CEBHI account is considered to achieve intergenerational equity when $|\Phi_{i}|\leq 0.2$ .

## 4 Parameter settings

The population parameters in this paper are primarily based on data from the Seventh National Population Census (hereinafter referred to as the “Seventh Census”), with a reference time of midnight on November 1, 2020. Consequently, the baseline time for calculations in this study is set at the end of 2020 or the beginning of 2021.

### 4.1 Insured population structure parameters

Using the cohort-component method, the urban–rural dual population iterative transfer model, and setting early 2021 as the baseline year, this paper projects urban and rural populations by age and gender over the forecast period. The CEBHI participation numbers are then derived by applying labor force participation and insurance coverage rates.

The initial population is derived from age- and gender-specific urban population data from the Seventh Census. Mortality and fertility rates are aligned with the medium-variant projections from the “World Population Prospects 2022” (24). Fertility rate differentials between urban and rural areas are maintained according to data from the 2020 Census. It is assumed that artificial gender selection will gradually diminish over the forecast period, with the infant sex ratio stabilizing at 107 by 2030. The urbanization rate is projected to increase steadily, reaching 75% by 2050. Throughout the forecast period, the demographic profile of rural-to-urban migrants will be consistent with that of rural residents registered outside their household registration locations, based on the 2020 Census data. Labor force participation rates are calculated from age- and gender-specific data on urban employment and total population from the Seventh Census. CEBHI coverage rates are projected to increase annually at an average of 1.3%, based on trends from the past 5 years, eventually reaching 90%.

### 4.2 Institutional parameters

The initial enrollment age for employees is set at 22, with a retirement age of 60 for men, an average retirement age of 52 for women (25, 26), and a maximum lifespan of 100 years. Participants are assumed to contribute continuously and in full from the start of employment until retirement. According to Document [1998] No. 44 issued by the State Council, the contribution rate for CEBHI is 8%, with 6% provided by employers and 2% by employees. Individual contributions and a portion of employer contributions are allocated to personal accounts, with the remainder directed to the pooled account. From 2018 to 2022, pooled income constituted an average of 61% of total fund income.

Document [2021] No. 14 from the General Office of the State Council reformed the allocation of personal accounts, mandating that all employer contributions be directed to the pooled fund. This paper assumes that the employer contribution rate of 6% will be fully transitioned to the pooled fund from 2022 to 2024, with complete allocation starting in 2025 (19). Additionally, it is assumed that retirees’ personal accounts will be credited at a fixed rate of 2% of the average basic pension level for the year, with pension growth expected to align with the economic growth rate.

### 4.3 Economic parameters

“The Comprehensive Plan for Reducing Social Insurance Contribution Rates,” issued by the General Office of the State Council and implemented on May 1, 2019, revised the calculation basis for the average wage of employed persons. This policy stipulates that the upper and lower limits of individual social insurance contribution bases should be determined based on the average salary of all urban employed persons. Accordingly, this paper uses the 2020 average wage for urban employed persons, 72,560 yuan, as the contribution base for the CEBHI. Based on the “Research Group on the Third Survey on the Social Status of Women in China” (27), women’s wages are assumed to be 70% of men’s wages. Using the average wage and employment data, the contribution base is 82,842.49 yuan for men and 57,989.74 yuan for women.

This study integrates methodological approaches from existing literature (28) and the U. S. Congressional Budget Office’s (29) phased growth framework, augmented by the Social Security Administration’s 1.63% baseline projection (30). We accordingly calibrate a sequential economic growth trajectory: 5.5% during 2023–2030, 4.5% for 2031–2040, 3.5% across 2041–2050, and 2.0% for post-2051 horizons. The wage growth rate moves in tandem with the economic growth rate.

The determination of discount rates must account for the uncertainty characteristics of capital. Theoretically, risk-free funds should be discounted using the risk-free term structure; however, due to the absence of full-maturity index bonds in most countries, scholarly practice typically calibrates discount rates within the range between the real return on short-term government bonds and private capital returns (23, 31). Empirical evidence reveals pronounced time-varying characteristics in discount rate selection: earlier studies predominantly adopted 5–6% (23), whereas contemporary research consistently converges toward approximately 3.5% (31). Anchored in the global low-interest-rate environment and China’s 10-year government bond average yield of 2.8%, this study employs a 3.5% discount rate for benchmark regression analyses.

### 4.4 Health insurance reimbursement-related parameters

#### 4.4.1 *Per capita* health insurance reimbursement

In 2020, the CEBHI pooled fund expenditure totaled 793.1 billion yuan, covering 344.55 million participants, with a per capita expenditure of 2,301.84 yuan. In subsequent calculations, this figure is used as the baseline for per capita health insurance reimbursement.

#### 4.4.2 Health insurance reimbursement weights by age group

As shown in Equation 4, estimating the present value of participants’ total lifetime health insurance reimbursement requires determining the health insurance reimbursement weights $\lambda_{i,t}$ for each age group by gender.

This paper employs a Generalized Linear Model (GLM) and a Mixed Linear Model (MLM) to assign health insurance reimbursement weights across different age groups to ensure accuracy. First, recognizing that medical expenses typically exhibit a positive mean, right skewness, and heavy tails (32), this paper assumes that reimbursement (Y) follows a gamma distribution with a log link function and uses the GLM to estimate age-specific reimbursement parameters. Second, to address the substantial number of zero values in reimbursement, this paper adopts the method proposed by Feng et al. (33) for handling medical expenses. The average reimbursement is calculated by gender and age group for insured employees each year. This pooled cross-sectional dataset is then used to estimate the parameters for per capita reimbursement by age group using ordinary least squares.

In both methods, the dependent variable is health insurance reimbursement. The main independent variables include age group age.i (specifically, age.1 to age.6, representing the age groups 20–29, 30–39, 40–49, 50–59, and 60–69, while those aged 70 and above are the reference group, not listed separately) and gender. The logarithmic form of income ( ln(income) ) is added as a control variable (34).

This paper utilizes data from the China Family Panel Studies (CFPS, 2014–2018) for empirical analysis, with the regression results presented in Table 1.

**Table 1:** Health insurance reimbursement regression results by mode.

Variable	GLM	MLM
Age.1	−2.0707***	−3.1401***
Age.2	−1.5483***	−2.4927***
Age.3	−1.1358***	−2.0407***
Age.4	−0.7228***	−1.1813***
Age.5	−0.4233***	−0.6512***
Gender	−0.1115	−0.4643**
Ln (income)	0.0061	0.5868
observations	3,791	11,946

*** *p*<0.01, ** *p*<0.05, * *p*<0.1.

As shown in Table 1, regression results from both methods indicate that each age group significantly impacts health insurance reimbursement. The coefficients for all age groups are negative, as the reference group consists of participants aged 70 and above, who receive the highest health insurance reimbursement. Notably, the MLM results reveal a significant effect of gender on health insurance reimbursement. This effect arises from the averaging process in MLM, which incorporates gender differences in disease prevalence into the health insurance reimbursement data. Year effects are controlled in both models.

Using the health insurance reimbursement parameters derived from the GLM, the reimbursement weights by gender and age are calculated according to Equation 11.


(11) $$\lambda_{i,j}=\frac{N\cdot \chi_{j}\cdot \mathrm{il}l_{i,j}}{\sum_{i=0}^{1}\sum_{j=1}^{6}\chi_{j}\cdot \mathrm{il}l_{i,j}\cdot n_{i,j}}$$

Where i ∈ {0,1}, with i = 0 representing females and i = 1 representing males; j ∈ {0,1,2,3,4,5}, representing different age groups; $\chi_{j}$ is the parameter for reimbursement, where $\chi_{j}=\exp(c.{\mathrm{age}}_{j})$ , and $c.{\mathrm{age}}_{j}$ denotes the coefficient for each age group in the regression results; $\mathrm{il}l_{i,j}$ represents disease prevalence; $\lambda_{i,j}$ is the reimbursement weight; $n_{i,j}$ is the sample size for each group, and N is the total sample size.

Using the health insurance reimbursement parameters derived from the MLM, the reimbursement weights by gender and age are calculated according to Equation 12.


(12) $$\lambda_{i,j}=\frac{N\cdot \chi_{i,j}}{\sum_{i=0}^{1}\sum_{j=1}^{6}\chi_{i,j}\cdot n_{i,j}}$$

Since gender has a significant effect on health insurance reimbursement in the MLM, the reimbursement parameter $\chi_{i,j}$ depends on both age group and gender. When *i* = 0, $\chi_{i,j}=\exp(c.{\mathrm{age}}_{j});$ when *i* = 1, $\chi_{\mathrm{ij}}=\exp(c.{\mathrm{age}}_{j})\cdot \exp(c.\text{gender}),$ where c.gender denotes the coefficient for gender in the regression results. The calculated results are presented in Table 2.

**Table 2:** Health insurance reimbursement weights.

Gender	Methods	Age 20–29	Age 30–39	Age 40–49	Age 50–59	Age 60–69	Age 70+
Male	GLM	0.145	0.290	0.421	0.825	1.622	3.135
	MLM	0.112	0.213	0.335	0.792	1.345	2.580
Female	GLM	0.170	0.342	0.528	1.060	1.646	3.325
	MLM	0.178	0.339	0.533	1.260	2.140	4.104

As shown in Table 2, the health insurance reimbursement weights calculated by these two methods are generally similar, with minor differences in two main aspects. First, the MLM assigns relatively higher reimbursement weights to older adults. Second, it demonstrates more pronounced gender differences in reimbursement weights. We adopt the weights calculated by the GLM as the baseline for age- and gender-specific health insurance reimbursement weights. In contrast, the weights from the MLM are used for robustness testing^1^.

#### 4.4.3 Growth rate of health insurance reimbursement

Considering the impact of population aging on the growth rate of health insurance reimbursement, this paper adjusts recent reimbursement data using calculated reimbursement weights to remove the effects of population aging. On this basis, the average annual growth rate of reimbursement is then calculated, as shown in Equations 13, 14.


(13) $${\bar{M}}_{k}=\frac{{\bar{M}}_{k}^{0}}{\nu_{k}}$$

(14) $$\nu_{k}=\sum_{\mathrm{ij}}\iota_{\mathrm{ijk}}\lambda_{\mathrm{ij}}$$

Where k denotes the year, $\nu_{k}$ is the adjustment factor, $\iota_{i,j,k}$ represents the population proportion, ${\bar{M}}_{k}^{0}$ indicates the per capita health insurance reimbursement, and ${\bar{M}}_{k}$ is the adjusted health insurance reimbursement. The meanings of the remaining symbols are consistent with prior definitions.

The estimated growth rates of health insurance reimbursement are presented in Table 3. Growth rates are derived from reimbursement weights calculated with the generalized linear model and mixed linear model, respectively.

**Table 3:** Health insurance reimbursement growth rates.

Methods	2015	2016	2017	2018	2019	2020	2021
GLM	8.33%	5.53%	9.03%	5.46%	11.21%	−6.83%	9.13%
MLM	8.12%	5.41%	8.91%	5.22%	11.15%	−6.92%	9.00%

Excluding 2020 and 2021, which were significantly impacted by the COVID-19 pandemic, the average annual GDP growth rate was 6.66%, while the average yearly growth rate of health insurance reimbursement reached 7.91% or 7.76%. Therefore, the average yearly growth rate of per capita reimbursement exceeded the GDP growth rate by 1.25% or 1.10%, reflecting a higher growth in reimbursement relative to the overall economy.

Accordingly, this paper assumes that the growth rate of per capita health insurance reimbursement exceeds the GDP growth rate by 1%.

## 5 Analysis of the generational actuarial balance and intergenerational equity of the CEBHI under the current policy

### 5.1 Baseline intergenerational accounting results and robustness tests

Building on the model and parameter settings, the paper employs MATLAB to develop an intergenerational accounting framework for the CEBHI system. Given that an insured individual aged 22 at the start of 2021 is projected to reach maximum lifespan by 2099, the fund account is assumed to achieve medium- to long-term actuarial balance beyond 2099.

Utilizing the intergenerational accounting system developed in this paper, the intergenerational account values for each generation under the baseline scenario were calculated precisely, and specific values for the corresponding generational actuarial balance and intergenerational equity indicators were derived. To ensure the robustness and reliability of the findings, robustness tests on the health insurance reimbursement weights were also conducted. All relevant results are presented in Table 4.

**Table 4:** Baseline intergenerational accounting results and robustness tests.

Indicator	Reimbursement	Current generation account	Future generation account	Generational actuarial balance	Intergenerational equity
Baseline	333,150.51	−174,781.82	378,546.39	−0.9515	3.0124
Robustness	340,340.63	−181,971.94	394,659.76	−0.9907	3.1393
Changes	2.16%	−4.11%	4.26%	4.12%	4.21%

The first row of Table 4 presents the intergenerational accounting results for the CEBHI system under the baseline scenario. The data reveal significant actuarial imbalance and intergenerational inequity in the system. Specifically, the health insurance benefits received by participants exceed their contributions, with an imbalance measure of 0.9515. Moreover, there is a substantial difference between future and current generations in the intergenerational accounting results, with the discrepancy amounting to 3.0124 times the contributions of the current generation. This reflects severe intergenerational inequity, whereby future generations are net contributors while the current generation is a net beneficiary.

The second and third rows of Table 4 show the robustness test results and corresponding changes. The changes are calculated as: (recalculated account value - baseline account value) / baseline account value. The generational actuarial balance or intergenerational equity indicator is calculated based on the absolute value of the recalculated and baseline values. The results indicate that, with this new reimbursement weight, reimbursement increases by 2.16%, the intergenerational account value for the current generation decreases by 4.11%, and that for future generations rises by 4.26%. Additionally, the generational actuarial balance and intergenerational equity indicators improve by 4.12 and 4.21%, respectively, with all changes within 5%. These findings suggest that the selected reimbursement weight in this study demonstrates a certain level of robustness.

### 5.2 Sensitivity testing

This section conducts sensitivity analysis through three critical dimensions: (1) examining the relationship between GDP growth rate and health insurance reimbursement growth rate, (2) scenario analysis with alternative discount rates, and (3) intergenerational burden of future generations under heterogeneous conditions.

#### 5.2.1 Testing of GDP-Reimbursement Growth Correlation

Based on the empirical findings of this study, after controlling for population aging factors, the growth rate of health insurance reimbursement is determined to exceed GDP growth by an average of 1 percentage point. This study establishes two policy scenarios: the optimistic scenario assumes health insurance reimbursement growth aligns with GDP growth, while the adverse scenario posits reimbursement growth exceeding GDP growth by 2 percentage points (14, 35). Table 5 presents the sensitivity test results under these two scenarios.

**Table 5:** The Impact of health insurance reimbursement growth rate on intergenerational accounts.

Health insurance reimbursement growth rate	Current generation account	Future generation account	Generational actuarial balance	Intergenerational equity
Optimistic scenario	−49,545.81	259,133.03	−0.2697	1.6805
Adverse scenario	−384,699.82	552,667.22	−2.0944	5.1032

Table 5 shows that under both optimistic and adverse scenarios, the CEBHI system exhibits actuarial imbalance and intergenerational inequity, validating the robustness of the baseline findings. Furthermore, the analysis reveals a critical relationship between medical reimbursement growth rates and CEBHI sustainability: when reimbursement growth aligns with GDP expansion (indicating optimal cost containment), actuarial imbalance and intergenerational inequity remain at lower levels. Conversely, when reimbursement growth exceeds GDP growth by 2 percentage points, both issues intensify significantly. These empirical findings demonstrate the essential role of medical cost control in maintaining long-term sustainability of the funds.

#### 5.2.2 Testing of discount rate variations

The baseline scenario adopts a 3.5% discount rate. Since the selection of discount rate directly influences the present value calculation of future cash flows, it substantially affects the measurement of intergenerational account values. Table 6 displays sensitivity test results under different discount rate scenarios.

**Table 6:** The impact of discount rate on intergenerational accounts.

Discount rate	Current generation account	Future generation account	Generational actuarial balance	Intergenerational equity
2%	−517,880.45	678,956.40	−2.1158	4.8898
2.5%	−384,699.82	552,667.22	−2.0944	5.1032
3%	−256,639.54	457,954.61	−1.2733	3.5455
4%	−114,378.76	314,162.82	−0.6813	2.5528
4.5%	−69,860.11	261,709.63	−0.4541	2.1551
5%	−37,126.92	218,773.66	−0.2626	1.8099

The results in Table 6 demonstrate that across all discount rate scenarios, the CEBHI system consistently exhibits actuarial imbalance and intergenerational inequity, confirming the robustness of the baseline findings. The study further reveals a clear intensification trend: as the discount rate decreases, both the actuarial deficit and the severity of intergenerational inequity escalate significantly. These findings carry critical policy implications. Against the backdrop of a persistently deepening global low-interest-rate environment, government authorities must adopt proactive measures to address the long-term sustainability challenges facing CEBHI funds.

#### 5.2.3 Testing of intergenerational burden heterogeneity via simulation

This study adheres to the standard practice in generational accounting literature by adopting the uniform burden assumption for future generations in the baseline scenario (23). To account for China’s incremental reform policy characteristics, the sensitivity analysis incorporates heterogeneity testing of intergenerational burdens through three policy scenarios: per-decade burden increments of 1, 5, and 10%, corresponding to gradual, moderate, and accelerated reform pathways, respectively. The projection outcomes under these scenarios are presented in Table 7.

**Table 7:** Intergenerational accounts under intergenerational burden heterogeneity.

Intergenerational burden heterogeneity	Current generation account	Future generation account	Generational actuarial balance	Intergenerational equity
1%	−174,781.82	385,804.27	−0.9515	3.0519
5%	−174,781.82	416,332.62	−0.9515	3.2181
10%	−174,781.82	457,790.87	−0.9515	3.4438

Table 7 demonstrates that under conditions of intergenerational burden heterogeneity, while the actuarial balance of the current generation remains stable, the intergenerational equity indicator shows gradual escalation, increasing to 3.0519, 3.2181, and 3.4438 when the burden-sharing ratio for future generations rises by 1, 5, and 10% per decade, respectively. This evidence systematically validates the robustness of baseline projections. The sustained upward trajectory of equity metrics further establishes a quantitative foundation for institutional optimization, underscoring the need for considering intergenerational burden allocation calculus in long-term policy design.

### 5.3 Heterogeneity analysis: public and private sector employees

This study is conducted within the framework of China’s social security system. It should be specifically noted that while the pension insurance system maintains separate systems for public sector institutions and enterprise employees, according to State Council Document [1998] No. 44, the CEBHI system implements uniform financing policies and fund management. This institutional characteristic is reflected in official statistics, where reports such as the National Basic Medical Security Development Statistical Bulletin consolidate insurance fund data from both sectors. Consequently, our baseline analysis adopts all CEBHI enrollees as the study cohort.

It should be specifically noted that significant disparities exist between the public and private sectors in terms of wage levels and pension benefits. These characteristics directly affect contribution bases and post-retirement personal account benefits. To comprehensively and accurately evaluate the operation of the CEBHI system, we conduct subgroup analyses of public and private sector employees, examining departmental differences in actuarial balance and intergenerational equity across different cohorts.

According to the China Statistical Yearbook 2023, the 2022 average wage of non-private sector employees served as the public-sector health insurance contribution base with a value of 114,029 yuan, while the corresponding figure for private-sector employees stood at 65,237 yuan. The China Labor Statistical Yearbook 2023 indicated that the per capita pension for retired civil servants and public institution personnel reached 73,198 yuan in the same year, compared to 37,783 yuan for enterprise retirees. Based on these key parametric differences, this study separately calculated the CEBHI generational accounts for the public and private sectors. The results are presented in Table 8.

**Table 8:** Public and private sector intergenerational accounts.

Sector	Current generation account	Future generation account	Generational actuarial balance	Intergenerational equity
Public Sector	−128,558.65	481,577.84	−0.5502	2.6110
Private Sector	−214,474.90	275,514.94	−1.6043	3.6652

Table 8 shows the current generational account values at −128558.65 yuan for the public sector and −214474.90 yuan for the private sector. This disparity reveals that current enrollees in both sectors are net beneficiaries of the CEBHI system, with the private sector’s net benefit surpassing the public sector’s by 86916.25 yuan. For future participants, the public sector’s generational account value rises to 481577.84 yuan, while the private sector’s reaches 275514.94 yuan, demonstrating that all forthcoming cohorts will transition to net contributors.

Further analysis using actuarial balance and intergenerational equity indicators reveals an actuarial balance of −0.5502 for the public sector and −1.6043 for the private sector, with intergenerational equity indicators measuring 2.6110 and 3.6625, respectively. This not only confirms intergenerational inequity in both sectors but also demonstrates that the inequity is structurally more severe in the private sector. The disparity is primarily attributable to the public sector’s higher contribution base.

## 6 Analysis of the impact of policy adjustments on generational actuarial balance and intergenerational equity

To enhance the sustainability of the CEBHI fund, Chinese government departments and certain pooled areas have implemented or are adjusting relevant policies based on empirical conditions. These policy adjustments include delaying the retirement age, proposing contributions from retirees, increasing contribution rates, and reducing reimbursement rates. This section employs scenario analysis to assess the potential impacts of these future policy changes on the generational actuarial balance and intergenerational equity of the CEBHI. Achieving generational actuarial balance and intergenerational equity is essential for fundamentally ensuring the long-term sustainability of the CEBHI fund.

### 6.1 Delaying the retirement age

“Decision of the Standing Committee of the National People’s Congress on Implementing a Gradual Delay of the Statutory Retirement Age” (September 2024), operating in concert with “Outline of the 14th Five-Year Plan and Long-Range Objectives Through 2035,” establishes China’s delayed retirement policy reform effective January 1, 2025, progressively extending statutory retirement ages from 60 to 63 years for male workers, 50 to 55 years for female workers, and 55 to 58 years for female cadres through differentiated implementation schedules: males and female cadres will undergo monthly postponement increments phased over four-month intervals while female workers experience accelerated adjustment achieving equivalent monthly delay every 2 months.

To compare the impacts of different delayed retirement schemes on the sustainability of the CEBHI Fund, evidence from the OECD (2023) indicates that among 38 OECD member countries, 23 have implemented retirement age postponement policies (36). The current average statutory retirement age for males is 64.4 years, while the projected retirement age for new labor market entrants will further increase to 66.3 years. Colombia maintains the lowest statutory retirement age threshold at 62 years, and Denmark sets the highest standard at 70 years. Building on this international experience and relevant literature (37), this study designs two progressive delayed retirement alternatives: the first scheme implements a one-month delay every 6 months, and the second adopts a 1-month delay every 4 months. Both scenarios assume implementation for all male and female employees starting in 2025, with the unified goal of raising the statutory retirement age to 65 years. Thus, three retirement delay schemes are used for a comprehensive analysis of the impact of the delayed retirement policy on the intergenerational account measurement system of CEBHI. The calculated results are presented in Table 9.

**Table 9:** The impact of delaying the retirement age on intergenerational accounts.

Delay plans	Indicator	Current generation account	Future generation account	Generational actuarial balance	Intergenerational equity
Plan 1	Value	−154,365.93	358,652.19	−0.7688	2.5449
	Changes	11.68%	−5.20%	−19.20%	−15.52%
Plan 2	Value	−145,005.40	355,437.99	−0.6945	2.3970
	Changes	17.04%	−6.10%	−27.01%	−20.43%
Plan 3	Value	−136,909.69	347,141.95	−0.6362	2.2492
	Changes	21.67%	−8.30%	−33.14%	−25.34%

Table 9 shows that the upcoming retirement age policy to be implemented by the Chinese government effectively enhances the generational actuarial balance and intergenerational equity of the CEBHI system. After implementing this policy, the value of the existing intergenerational accounts increased by 11.68%, while the value of future generations’ intergenerational accounts decreased by 5.2%. This change resulted in a 19.20% improvement in generational actuarial balance and a 15.52% improvement in intergenerational equity. However, if delayed retirement policies 2 or 3 were implemented, the improvements in generational actuarial balance and intergenerational equity would be even more significant.

### 6.2 Retiree contribution

The CEBHI system was established based on the labor protection system. Under the labor protection system, retirees are not required to pay premiums for health insurance. Additionally, when the CEBHI was established, the wages of retirees in China were relatively low. A policy of exempting retirees from contributions was adopted to ensure the smooth implementation of the CEBHI system. Since 2015, various documents, including the Fifth Plenary Session of the 18th Central Committee of the Communist Party of China, the national “13th Five-Year Plan” outline, and Beijing’s “13th Five-Year” human resources and social development plan, have proposed to improve the mechanisms for stable and sustainable funding and reimbursement ratio adjustments for health insurance, and to study the implementation of a health insurance contribution policy for retired employees.

There are two primary justifications for proposing that retirees should contribute to health insurance: first, mandating contributions from retirees can enhance the sustainability of the health insurance fund; second, in countries with established social health insurance systems, it is common for retirees to contribute throughout their lifetime (38).

Referring to the contribution rate settings for retirees in existing literature (39, 40), this paper assumes a maximum contribution rate of 5% for retirees and assesses the impact of retiree contributions on intergenerational accounts. To maintain the continuity of the calculation process, two additional scenarios are also analyzed: retirees making no contributions (with 1% of pension transferred from the pooled account to the individual account, i.e., a 1% contribution rate), and retirees making no contributions with no transfers (i.e., a 0% contribution rate), as presented in Table 10.

**Table 10:** The impact of retiree contribution rate on intergenerational accounts.

Retirement contribution rate	Indicator	Current generation account	Future generation account	Generational actuarial balance	Intergenerational equity
−1%	Value	−162,125.25	356,231.45	−0.8826	2.8220
	Changes	7.24%	−5.89%	−7.24%	−6.32%
0%	Value	−149,468.67	333,937.70	−0.8137	2.6318
	Changes	14.48%	−11.78%	−14.48%	−12.63%
1%	Value	−136,812.09	311,643.95	−0.7448	2.4415
	Changes	21.72%	−17.67%	−21.72%	−18.95%
2%	Value	−124,155.50	289,329.01	−0.6759	2.2511
	Changes	28.97%	−23.57%	−28.96%	−25.27%
3%	Value	−111,498.94	267,056.45	−0.6070	2.0609
	Changes	36.21%	−29.45%	−36.21%	−31.59%
4%	Value	−98,842.36	244,762.7	−0.5381	1.8707
	Changes	43.45%	−35.34%	−43.45%	−37.90%
5%	Value	−86,185.76	222,415.98	−0.4692	1.6801
	Changes	50.69%	−41.24%	−50.69%	−44.23%

From Table 10, the data indicate that increased contributions from retirees enhance the generational actuarial balance and intergenerational equity of CEBHI. A higher post-retirement contribution rate results in larger current intergenerational accounts and a reduced burden on future generations’ accounts, thereby promoting greater generational actuarial balance and intergenerational equity. For instance, with a post-retirement contribution rate of 2%, compared to the baseline scenario, current intergenerational accounts increase by 28.97%, future intergenerational accounts decrease by 23.57%, the generational actuarial balance improves by 28.96%, and intergenerational equity improves by 25.27%.

### 6.3 Increasing the contribution rate

Document [1998] No. 44 indicates that with economic development, employers’ contribution rates for CEBHI may be adjusted accordingly. In practice, some pooled regions have recorded employer contribution rates as high as 10%. This paper analyzes the changes in CEBHI intergenerational accounts at employer contribution rates of 7, 8, 9, and 10%, with the results presented in Table 11.

**Table 11:** The impact of contribution rates on intergenerational accounts.

Contribution rate	Indicator	Current generation account	Future generation account	Actuarial balance	Intergenerational equity
7%	Value	−144,551.67	359,339.93	−0.6758	2.3556
	Changes	17.30%	−5.07%	−28.98%	−21.80%
8%	Value	−114,321.52	340,133.47	−0.4683	1.8614
	Changes	34.59%	−10.15%	−50.78%	−38.21%
9%	Value	−84,091.38	320,927.02	−0.3065	1.4762
	Changes	51.89%	−15.22%	−67.79%	−51.00%
10%	Value	−53,861.23	301,720.56	−0.1768	1.1674
	Changes	69.18%	−20.29%	−81.42%	−61.25%

From Table 11, the data indicate that an increase in the contribution rate for CEBHI enhances the system’s generational actuarial balance and intergenerational equity. A higher contribution rate results in larger current intergenerational accounts and a reduced burden on future generations’ accounts, thus promoting greater generational actuarial balance and intergenerational equity. For instance, with a contribution rate of 7%, compared to the baseline scenario, the current intergenerational accounts increase by 17.30%, future intergenerational accounts decrease by 5.07%, the generational actuarial balance improves by 28.98%, and intergenerational equity improves by 21.80%.

### 6.4 Reducing the reimbursement rate

“According to the Statistical Bulletin on the Development of National Medical Security” (2018–2020), in the past 5 years, the highest reimbursement rate for the CEBHI was 62.15%, the lowest was 53.49%, and the average reimbursement rate was 58.01%. Based on this data, this article establishes the reimbursement rate at 58% for the baseline scenario. Document No. 44 [1998] issued by the State Council stipulates that the CEBHI system operates on a pay-as-you-go basis, alongside a management model predicated on revenue and expenditure. In this framework, as medical expenditures increase annually, the reimbursement rate may be adversely affected and decrease. However, to ensure policy stability, any reduction in the reimbursement rate should be maintained within a reasonable range. Therefore, to evaluate the potential impacts of a decreased reimbursement rate on the intergenerational accounts of the CEBHI, this article posits reimbursement rates of 55, 50, and 45% for further analysis.

As shown in Table 12, reducing the health insurance reimbursement rate enhances the generational actuarial balance and intergenerational equity of the CEBHI. A lower reimbursement rate results in larger current generation intergenerational accounts and diminishes the burden on future generations’ intergenerational accounts, thereby improving generational actuarial balance and intergenerational equity. For example, with a reimbursement rate of 55%, compared to the baseline scenario, the current generation intergenerational accounts increase by 14.06%, while the future intergenerational accounts decrease by 9%. Additionally, the generational actuarial balance improves by 14.05%, and intergenerational equity rises by 10.60%.

**Table 12:** The impact of reimbursement rate on intergenerational accounts.

Reimbursement rates	Indicator	Current generation account	Future generation account	Generational actuarial balance	Intergenerational equity
55%	Value Changes	−150,207.99 14.06%	344,488.41 −9.00%	−0.8178 −14.05%	2.6932 −10.60%
50%	Value Changes	−122,146.35 30.11%	305,596.53 −19.27%	−0.6650 −30.11%	2.3287 −22.70%
45%	Value Changes	−94,094.85 46.16%	266,718.68 −29.54%	−0.5123 −46.16%	1.9643 −34.79%

### 6.5 Comprehensive analysis of the impacts of policy changes

The preceding calculation results show that delaying retirement, retiree contributions, increasing contribution rates, and reducing health insurance reimbursement can improve the generational actuarial balance and intergenerational equity of the CEBHI intergenerational accounts. However, a single policy adjustment alone cannot achieve generational actuarial balance and intergenerational equity. Furthermore, the operation of the CEBHI system in practice is subject to the cumulative impact of multiple policy changes. Therefore, this paper conducts a simulation analysis of various policy combinations to propose feasible policy improvements for achieving generational actuarial balance and intergenerational equity in CEBHI intergenerational accounts.

Based on the feasibility of policy implementation, this paper evaluates the effects of 480 policy combinations. These combinations include three delayed retirement plans, with the contribution status of retired employees categorized into eight types: after retirement, the proportion of pension transferred from the pooled account to the individual account is 2% or 1%; the pooled account does not transfer and retirees make no contributions; and retired employees continue to contribute, with contribution rates set at 1, 2, 3, 4% or 5%. Additionally, the employer contribution rate is set at five levels: 6, 7, 8, 9, and 10%, while the health insurance reimbursement rate is divided into four levels: 58, 55, 50, and 45%.

**Figure 1:**
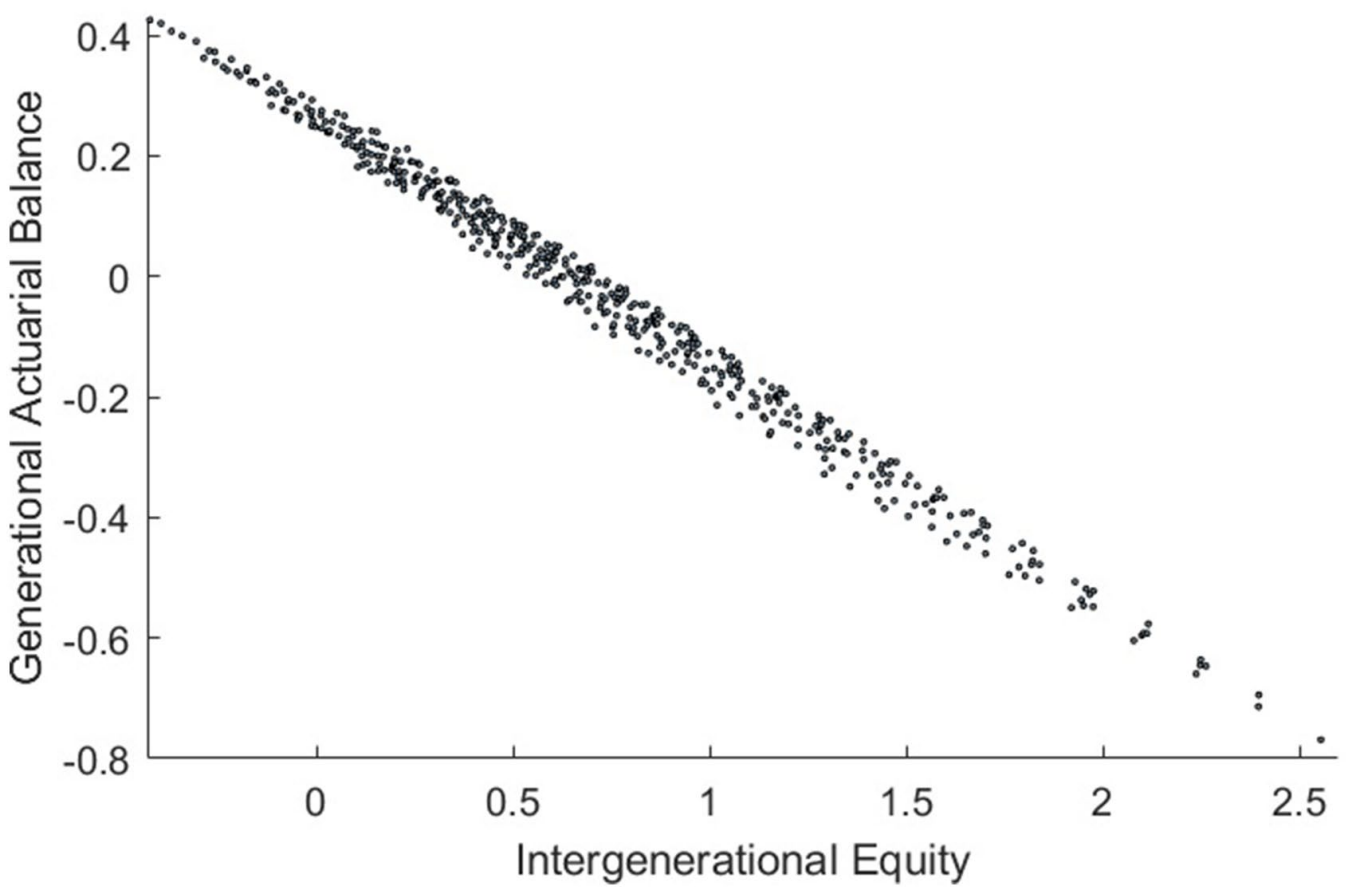
Comprehensive analysis of the impacts of policy change.

The generational actuarial balance and intergenerational equity indicators for the 480 policy combinations are shown in Figure 1. The range for generational actuarial balance is [−0.7688, 0.4259], while the range for intergenerational equity is [−0.4229, 2.5549]. Both ranges include zero, indicating that CEBHI can achieve either generational actuarial balance or intergenerational equity through adjustments in policy combinations. However, the ranges do not include the origin, as when the generational actuarial balance indicator equals zero, it reflects the balance between lifetime contributions and benefits for a 22-year-old insured individual at the beginning of 2021. In all other cases, most of the existing intergenerational accounts are negative, and future generations still bear a certain level of implicit debt, which is why the intergenerational equity indicator does not equal zero.

Based on the generational actuarial balance and intergenerational equity criteria, the corresponding indicators must be below 0.2—16 policy combinations out of 480 simultaneously satisfy both conditions. Policymakers can choose suitable policy combinations based on the feasibility of adjustments and intended objectives, making dynamic adjustments to ensure that the CEBHI system maintains generational actuarial balance and intergenerational equity, thereby supporting its sustainability.

It is important to note that only three of these 16 policy combinations are based on the delayed retirement age policy soon to be introduced by the Chinese government, while the remaining 13 require implementing policies with a longer retirement age. Furthermore, all 16 policy combinations necessitate introducing a retiree contribution policy. This highlights the need for further research on retirement age and retiree contribution policies in China to enhance the sustainability of the CEBHI system.

## 7 Conclusions and research limitations

### 7.1 Conclusion

This study addresses the sustainable risk of public health insurance schemes, explicitly focusing on China’s Employee Basic Health Insurance. It explores policy strategies to maintain the system’s sustainability from generational actuarial balance and intergenerational equity perspectives. This paper addresses two key issues: first, whether the current CEBHI system meets the requirements for generational actuarial balance and intergenerational equity; second, how policy adjustments can ensure the achievement of both, thereby securing the fund’s long-term sustainability.

This paper combines actuarial models and generational accounting to establish a generational account system, defining generational actuarial balance and intergenerational equity indicators under the fund’s sustainability constraints. With reasonable parameter settings, the results show significant generational actuarial imbalance and intergenerational inequity in the current CEBHI system.

Scenario analysis is used to evaluate the impact of policy adjustments on the fund. Delaying retirement, requiring retirees to contribute, increasing contribution rates, and reducing reimbursement rates all mitigate actuarial imbalance and intergenerational inequity. However, no single policy can achieve both. Comprehensive analysis reveals an inverse relationship between generational actuarial balance and intergenerational equity indicators, as many generational accounts are negative, and current and future generations share the resulting implicit debt.

The findings of this study provide important policy implications. First, policy coordination is essential to achieving generational actuarial balance and intergenerational equity in CEBHI. Adjustable policies include delaying the retirement age, requiring retirees to contribute, increasing contribution rates, and reducing reimbursement rates. Depending on the policy implementation context and the feasibility of adjustments, an appropriate policy mix should be selected to synergistically promote generational actuarial balance and intergenerational equity in CEBHI, thereby providing dynamic adjustment solutions and references for ensuring the sustainability of the CEBHI fund.

Second, the comprehensive analysis of policy reforms reveals structural limitations in the Chinese government’s implementation of delayed retirement policies, which are insufficient to achieve the dual objectives of actuarial balance and intergenerational equity independently of the CEBHI system. This study’s actuarial projections indicate that maintaining the actuarial balance and intergenerational equity requires synchronized reinforcement of both delayed retirement policies and retiree contribution mechanisms, as these measures are pivotal in all viable policy portfolios. However, their implementation will incur substantial societal adaptation costs: insured individuals will face forced extensions to their working years with corresponding reductions in retirement duration, while direct deductions of insurance contributions from pension benefits will erode retirees’ disposable income. These findings underscore the necessity for the Chinese government to conduct in-depth research on retirement age adjustments and retiree contribution policies, balancing the long-term sustainability of the CEBHI fund with rigorous evaluations of societal affordability.

### 7.2 Research limitations

This study has several limitations that need to be acknowledged.

First, at the methodological level, the analysis relies on aggregated national data without sufficiently controlling for regional heterogeneity such as localized wage levels and enrollment rates. This may lead to deviations between nationwide actuarial projections and localized operational realities. Currently, only eight provinces in China—including the four direct-administered municipalities—have achieved province-wide pooling of CEBHI system (41). This fragmented management structure could result in systematic discrepancies between national-level estimates and ground-level implementation outcomes.

Second, variations in social insurance contribution compliance were excluded from the modeling framework. In practice, enterprises exhibit heterogeneous contribution behaviors—from full-wage compliance to minimum-threshold remittances—resulting in an aggregate compliance rate of 51.30% (42). This empirical reality may substantially bias generational accounting projections.

Finally, while this study validates pathways for enhancing the sustainability of the CEBHI through policy scenario simulations, it fails to comprehensively assess the impact of policy adjustments on all affected entities. This includes potential welfare effects on distinct groups such as active employees, retirees, companies, and local governments. These underexplored dimensions establish critical priorities for subsequent health policy research.

## Data Availability

The raw data supporting the conclusions of this article will be made available by the authors, without undue reservation.

## Footnotes

^1^This study further calculates gender-specific outpatient expenditure reimbursement weights across age cohorts. The results demonstrate that older adults patients receive significantly higher health insurance reimbursement weights for outpatient services compared to the universal coverage scheme (detailed calculations in the Supplementary material). While State Council Document No.14 (2021) requires a policy tilt toward retirees, this approach worsens CEBHI’s actuarial imbalance and intergenerational inequity under current policies.
