# Supplementary material for: Address sustainability risks in health insurance funds: generational actuarial balance and intergenerational equity perspective
Source: Front Public Health. 2025 Aug 8;13:1641233. doi: 10.3389/fpubh.2025.1641233 (PMC12370485; doi:10.3389/fpubh.2025.1641233)
Supplement: Supplementary file 1 [file Data_Sheet_1.pdf]

Supplementary Material

Address Sustainability Risks in Health Insurance Funds: Generational Actuarial Balance and Intergenerational Equity Perspective

Yi Qin[1*], Wenfang Ji[2]

(Manuscript ID: 1641233)

Supplementary Table 1 presents the generalized linear model (GLM) and mixed linear model (MLM) regression results for health insurance reimbursement of outpatient costs.

Supplementary Table 1.

Variable	GLM	MLM
Age.1	-2.3109***	-3.1962***
Age.2	-2.0293***	-2.7274***
Age.3	-1.8582***	-2.7135***
Age.4	-0.9294***	-1.4740***
Age.5	-0.3484***	-0.6343**
Gender	-0.1464	-0.9054**
Ln(income)	0.0009	0.2785
observations	3512	11946

*** p<0.01, ** p<0.05, * p<0.1

Based on the reimbursement weight calculation formula in this study, the health insurance reimbursement weights for outpatient costs were derived. The results are presented in Supplementary Table 2.

Supplementary Table 2. Health insurance reimbursement weights.

Gender	Methods	Age 20-29	Age 30-39	Age 40-49	Age 50-59	Age 60-69	Age 70+
Male	GLM	0.135	0.193	0.200	0.599	1.981	3.444
	MLM	0.058	0.093	0.094	0.326	0.754	1.422
Female	GLM	0.135	0.208	0.238	0.931	2.009	3.539
	MLM	0.144	0.230	0.233	0.806	1.865	3.517

As demonstrated in Supplementary Table 2, the medical insurance reimbursement weights for outpatient expenditure among elderly patients are significantly higher than those under the universal coverage scheme.
